# The effect of the menstrual cycle and hyperglycaemia on hormonal and metabolic responses during exercise

**DOI:** 10.1007/s00421-021-04754-w

**Published:** 2021-07-08

**Authors:** A. T. Hulton, J. J. Malone, I. T. Campbell, D. P. M. MacLaren

**Affiliations:** 1 Department of Nutritional Sciences, School of Biosciences and Medicine, Faculty of Health and Medical Sciences, University of Surrey, Guildford, UK; 2 School of Health Sciences, Liverpool Hope University, Liverpool, UK; 3 Dept of Anaesthesia, Wythenshawe Hospital, Manchester, UK; 4 Research Institute for Sport and Exercise Sciences, Liverpool John Moores University, Liverpool, UK; 5 Sport and Exercise Science, University of Surrey, Leggett Building, Guildford, GU2 7WG UK

**Keywords:** Glucose infusion, Moderate exercise, Estrogen, Progesterone, Follicular, Luteal

## Abstract

**Purpose:**

Variations in substrate metabolism have been identified in women during continuous steady-state aerobic exercise performed at the same relative intensity throughout discrete phases of the menstrual cycle, although some evidence exists that this is abolished when carbohydrate is ingested. This investigation examined the effects of a supraphysiologic exogenous glucose infusion protocol, administered during two phases of the menstrual cycle (follicular and luteal) in eumenorrheic women to identify differences between metabolic, hormonal and substrate oxidative responses.

**Methods:**

During the experimental conditions, blood glucose was infused intravenously at rates to “clamp” blood glucose at 10 mM in seven healthy females (age 20 ± 1 y, mass 55.0 ± 4.1 kg, $\dot V{O_{2peak}}$ 40.0 ± 1.8 ml/kg/min). Following 30 min of seated rest, participants exercised on a cycle ergometer for 90 min at 60% $\dot V{O_{2peak}}$. During the rest period and throughout exercise, blood metabolites and hormones were collected at regular intervals, in addition to expired air for the measurement of substrate oxidation.

**Results:**

Significant differences between ovarian hormones and menstrual phase were identified, with estrogen significantly higher during the luteal phase compared to the follicular phase (213.28 ± 30.70 pmol/l vs 103.86 ± 13.85 pmol/l; *p = *0.016), and for progesterone (14.23 ± 4.88 vs 2.11 ± 0.36 nmol/l; *p = *0.042). However, no further significance was identified in any of the hormonal, metabolite or substrate utilisation patterns between phases.

**Conclusion:**

These data demonstrate that the infusion of a supraphysiological glucose dose curtails any likely metabolic influence employed by the fluctuation of ovarian hormones in eumenorrheic women during moderate exercise.

## Introduction

The main energy substrates used during endurance exercise are carbohydrates (CHO) and fats. The contribution of carbohydrate or fat to energy depends on exercise intensity, mode and duration, nutrition and training status, age, and sex, and these factors should be taken into account when substrate metabolism is analysed. In spite of the existing knowledge, data sources are few and there are conflicting findings regarding substrate metabolism in females (Kraemer et al. 2013; Oosthuyse et al. 2005; Vaiksaar et al. 2011). The majority of studies have observed that females rely on more fat as a primary substrate during exercise than men (Horton et al. 1998; Impey et al. 2020; Tarnopolsky^2000^; Wiecek et al. 2017), and these investigations invariable select the mid-follicular phase when undertaking such studies.

The ovarian hormones not only regulate the female menstrual cycle, but they also exert significant metabolic effects. It is this fact that accounts for variations between males and females with regard to substrate oxidation (Oosthyse and Bosch 2010). Estrogens promote elevated muscle glycogen synthesis activity, and so promote glycogen storage (Constantini et al. 2005). Estrogens also stimulate lipolysis and increase the availability of plasma-free fatty acids (FFA) during prolonged exercise (Dawson and Reilly, 2009; Oosthuyse and Bosc, 2010; Hackney, 1999; Tarnopolsky et al. 1995). In contrast, progesterone is understood to counteract many of the effects of estrogen (Hatta et al. 1988). Levels of progesterone are 12–20 times greater during the luteal phase than during the follicular phase, whereas estrogen is only 3 times greater (Landgren et al. 1980). Hence, varying levels of these hormones throughout the menstrual cycle may alter skeletal muscle metabolism during exercise.

Previous studies investigating exercise metabolism in different phases of the menstrual cycle have produced equivocal results. Several studies investigating short-term or intermittent exercise have found no differences between the mid-follicular and the late-luteal phase (Lynch and Nimmo 1998). Additionally, some studies have found no phase effect on metabolism during prolonged exercise (Bailey et al. 2000; Kanaley et al. 1992), whereas a few studies have found that the menstrual cycle phase does affect hormonal and metabolic response to exercise (Hackney 1999; Nicklas et al. 1989). Notably, these changes relate to greater lipid oxidation and reduced muscle glycogen use during the mid-luteal than mid-follicular phase, and when exercise is conducted in a CHO-depleted nutritional state these phase differences are more noticeable (Lavoie et al. 1987).

Exogenous CHO feeding has been observed to influence CHO oxidation in an age-dependent manner with adolescent females. Timmons et al. (2007) provided exogenous CHO and observed greater endogenous CHO oxidation in pubescent females who had higher levels of serum estrogen compared to pre-pubescent females. The variations in CHO oxidation are not observed during childhood, and only express themselves at the onset of puberty, once sex hormones impact substrate metabolism, transport and utilisation (Aucouturier et al. 2008). Campbell et al. (2001) observed an attenuated response of % contribution of CHO to total oxidation during 2-h cycling in the luteal compared with follicular phase when participants were provided with placebo, but that there was no difference when glucose was ingestion (~ 110 g CHO in 2-h). The improvements in time to complete 4 kJ/kg body weight of performance after the 2-h cycle exercise were noted for CHO feedings in comparison with placebo for both phases of the menstrual cycle, although there was a 13% faster time in the follicular phase compared to the luteal with placebo (24:30 ± 2:07 min vs. 28:17 ± 3:13 min). No difference was evident when CHO was ingested. So, it appears that CHO oxidation is favoured during the follicular phase, which is in contrast to the luteal phase which favours fat oxidation. These variations disappear when CHO feeding is implemented.

It is possible to take an extreme scenario vis a vis CHO or fat use during exercise by promoting hyperglycaemia using infusion of glucose. The effect of maintained hyperglycaemia during exercise, to understand hormonal and metabolic responses during an extreme/supraphysiologic exogenous CHO delivery protocol, has previously been studied with results indicating a diminished fatty acid and glycerol concentration with an increase in the rate of CHO oxidation (MacLaren et al. 1999; Malone et al. 2019; Mohebbi et al. 2020). The previous literature has been undertaken exclusively with male exercising participants, and to the authors' knowledge, there are no such studies employing female participants whilst exercising. However, an earlier study (Diamond et al. 1989) using the hyperglycaemic clamp at rest during two phases of the menstrual cycle found glucose metabolism to be impaired in the mid-luteal phase of the menstrual cycle. This appears to contradict the observation that CHO feeding leads to similar metabolic responses between the phases, albeit this was reported during exercise.

These findings raise questions as to the importance of the menstrual phase due to the variations of sex hormones throughout the cycle, and their potential effect on the hormonal and metabolic responses during exercise. Therefore, the aims of this study were to examine glucose utilization in eumenorrheic women during maintained hyperglycaemia throughout submaximal endurance exercise (60% $\dot V{O_{2peak}}$) and to examine the interrelationship between the ovarian hormones, metabolic hormones and substrate utilization. We hypothesized there will be no significant differences between the follicular and the luteal phase regarding hormonal and metabolic responses during moderate exercise due to the maintained hyperglycaemic conditions.

## Methodology

### Participants

Seven recreationally active and healthy females (age 20.1 ± 0.9 y, mass 55.0 ± 4.1 kg, $\dot V{O_{2peak}}$ 40.0 ± 1.8 ml/kg/min) provided informed consent in accordance with the procedures approved by the Ethics Committee of Liverpool John Moores University. Participants did not have any family history of diabetes, were not taking any form of hormonal contraception, and had regular menstrual cycles. Prior to participation in the study, each participant’s menstrual cycle was ‘tracked’ for 3 months. Each participant had to complete three months of menstrual cycle feedback before engaging in the project and were given The ClearPlan Easy™ Fertility Monitor kits for verification. Participants completed the Activity Readiness Questionnaire and underwent a brief physical examination performed by the physician to be approved to enter this investigation.

### Experimental design

This investigation was completed with a repeated measures design, with participants randomly assigned to undertake the investigation in a specific phase first. The hyperglycaemic glucose clamp technique was then employed during two separate phases during a normal menstrual cycle, once during the follicular phase (when ovarian hormones are low) and once during the luteal phase (when ovarian hormones are elevated). Blood glucose was elevated to 10 mmol/L^−1^ during 30 min of seated rest and then maintained at 10 mmol/L^−1^ during 90 min of exercise on a cycle ergometer at 60% $\dot V{O_{peak}}.$ Regular blood samples were taken for analysis of metabolic hormones and metabolites, in addition throughout the protocol expired air was collected and analysed for the determination of substrate utilisation.

### Experimental protocol

Participants attended the laboratory on three occasions. The first session involved a $\dot V{O_{2peak}}$ test whereby the participant exercised on a cycle ergometer (Cybex, Met 100: Rankonkrane, New York, USA) at an initial workload of 150 Watts, which increased by 30 Watts every 2 min until volitional exhaustion. For the assumption that $\dot V{O_{2peak}}$ was achieved, heart rate values of 10 ± participants age-predicted max and a rating of perceived exertion of  > 17 were recorded (ACSM 2010). Throughout this procedure, $\dot V{O_2}$ was measured using an online gas analysis system (MedGraphics, St Paul, MN, USA) for which the second minute mean of each exercise workload was calculated and used to calculate the $\dot V{O_2}$-workload regression equation. The mean $\dot V{O_2}$ value obtained during the final 30 s was calculated as the $\dot V{O_{2peak}}$ measurement. Using the regression equation, the workload representing 60% $\dot V{O_{2peak}}$ was calculated.

The following two occasions were conducted during the specific phase within the menstrual cycle, once during their follicular phase (days 3–10) and once during their luteal phase (days 6–11 post-ovulation). Participants were assigned a condition order randomly within a cross-over design. To ensure ovulation had occurred, participants were given ovulation testing kits (Clearplan, Unipath, Bedford, England) that detect luteinising hormone in the urine. These kits were used every morning from day 10 until ovulation was detected.

Participants fasted for 12 h prior to the investigation, but abstained from caffeine, alcohol and strenuous exercise for 24 h prior to testing. Furthermore, participants ingested the same diet in the 24 h prior to testing in both phases. Participants arrived at the laboratory at 08:00 whereupon weight was recorded. After the participants had voided urine they lay supine while a 16-guage iv cannula was inserted into the forearm vein of the left hand under a local anaesthetic. The right hand was placed in a hotbox set at 70 °C to arterialise the blood, and after 20 min another 16-guage cannula was inserted retrogradely into the forearm vein for blood sampling. Slow infusion of 0.9% saline was used to maintain patency of the right-hand cannula. The participants then rested for 20 min seated on the cycle ergometer before a 20-ml resting blood sample was taken. A priming infusion (Colleague, Baxter, Northampton, England) of 20% dextrose was then initiated into the left-hand vein to increase the plasma glucose concentration to 10 mmol/L^−1^, in accordance with the method outlined by DeFronzo et al. (1979). Participants remained seated at rest on the cycle ergometer for 30 min once the infusion was started, whereupon a pre-exercise, post-infusion 15 ml blood sample was taken before the initiation of cycling exercise at 60% $\dot V{O_{2peak}}$. The exercise was maintained for 90 min during which the plasma glucose concentration was maintained at 10 mmol/L^−1^ by varying the rate of glucose infusion every five minutes. This was achieved by taking a small volume of blood every five minutes for the determination of glucose concentration (Hemocue, Angelholm, Sweden). Glucose utilization rate (GUR) and the M/I ratio (the mean GUR divided by the mean insulin concentration), a measure of β-cell sensitivity to glucose, were calculated. Throughout exercise, the participants ingested 150 ml of water every fifteen minutes.

Blood samples (15 ml) were taken at 15, 30 and 60 min of exercise with another 15 ml sample taken at 90 min. The 15 ml blood sample was aliquoted into two 4-ml serum gel tubes for the analysis of progesterone, estrogen, insulin, cortisol, growth hormone (HGH), glycerol, non-esterified fatty acids (NEFA) and lactate. All blood samples were centrifuged at 3000 rpm at 3 °C for fifteen minutes and the serum stored at − 80 °C. Whole blood was also immediately analysed for haemoglobin and hematocrit levels.

Throughout the session expired air was analysed for 5-min periods pre-infusion, pre-exercise and at 15, 30, 60 and 90 min of exercise. This was conducted using an online gas analysis system (MedGraphics, St.Paul, MN, USA), and $\dot V{O_2}$ and RER values were used for the determination of substrate oxidation rates using calculations by Frayn et al. (1983). Heart rate was measured pre-infusion whilst in the supine position, prior to the start of exercise (0 min), then during exercise at 15, 30, 60, 90 min using a PE300 Heart Rate Monitor (Polar Electropolar OY, Kempele, Finland).

Participants repeated the protocol not less than 2-weeks later depending on the length of their menstrual cycle. During the second testing session, the glucose was infused into the other hand to decrease any risk of thrombosis.

### Biochemical analyses

The hormonal analyses for progesterone, estrogen, insulin, cortisol, and human growth hormone were performed by ELISA using the Anthos HTII microplate reader and employing appropriate standardised kits from DRG Instruments GmbH (Germany). The intra-assay CV for the assays were 3.0% for insulin, 3.6% for human growth hormone, 2.2% for cortisol, 5.7% for estrogen, and 6.8% for progesterone. All hormonal analysis was measured in duplicate with mean value provided.

Plasma NEFA values were determined by an enzymatic spectrophotometric method, while a portion of the plasma was deproteinized with perchloric acid (7% wt/vol) before assay for lactate and glycerol using enzymatic methods. Analyses were performed on a Cobas-Bio centrifugal analyser (Roche Products, Welwyn Garden City, Herts, UK). Plasma insulin was determined using radioimmunoassay (RIA) (IM.78, Amersham International, Amersham, UK).

### Statistics

SPSS software (version 25 SPSS, Chicago, IL) was used for data entry and analysis for all measurements. Data normality was assessed using Shapiro–Wilks tests, and due to normality, meta-analysis of variance for repeated measures was employed to determine significant differences between the two phases (follicular and luteal) and time points for plasma metabolites, hormones and substrate oxidation rates. Significant p-values were then examined further using the post hoc Tukey test. Estrogen and progesterone were analysed between phases using a paired *t* test. Effect size was calculated using Cohen’s d effect size value calculation (Cohen 1969) and 95% confidence intervals were displayed for significant findings. Data are expressed as mean ± SEM with alpha significance accepted at the *p < *0.05 level.

## Results

### Changes in plasma volume

Differences in plasma volume changes between phases were 3.6% and 1.2% between follicular and luteal but not significant. Therefore, metabolite data were not corrected.

### Performance and blood metabolites

Mean heart rate was not affected by the glucose infusion at rest although exercise significantly increased heart rate from 81 ± 6 bpm to 165 ± 4 beats.min^−1^ (*p* < 0.01) during the first 15 min. There was no significant change in heart rate over the remaining exercise period, and no significant differences between the phases.

There were no significant differences in pre-infusion blood glucose concentration between the phases, with mean values of 4.73 ± 0.09 mmol/l and 4.63 ± 0.14 mmol/l for the follicular and luteal phases, respectively. Glucose infusion resulted in a maintained blood glucose concentration of 10.10 ± 1.08 mmol/l during the follicular phase and 10.15 ± 1.50 mmol/l during the luteal phase, with no significant difference between the two phases (*p* > 0.05) throughout the exercise infused period.

**Fig. 1:**
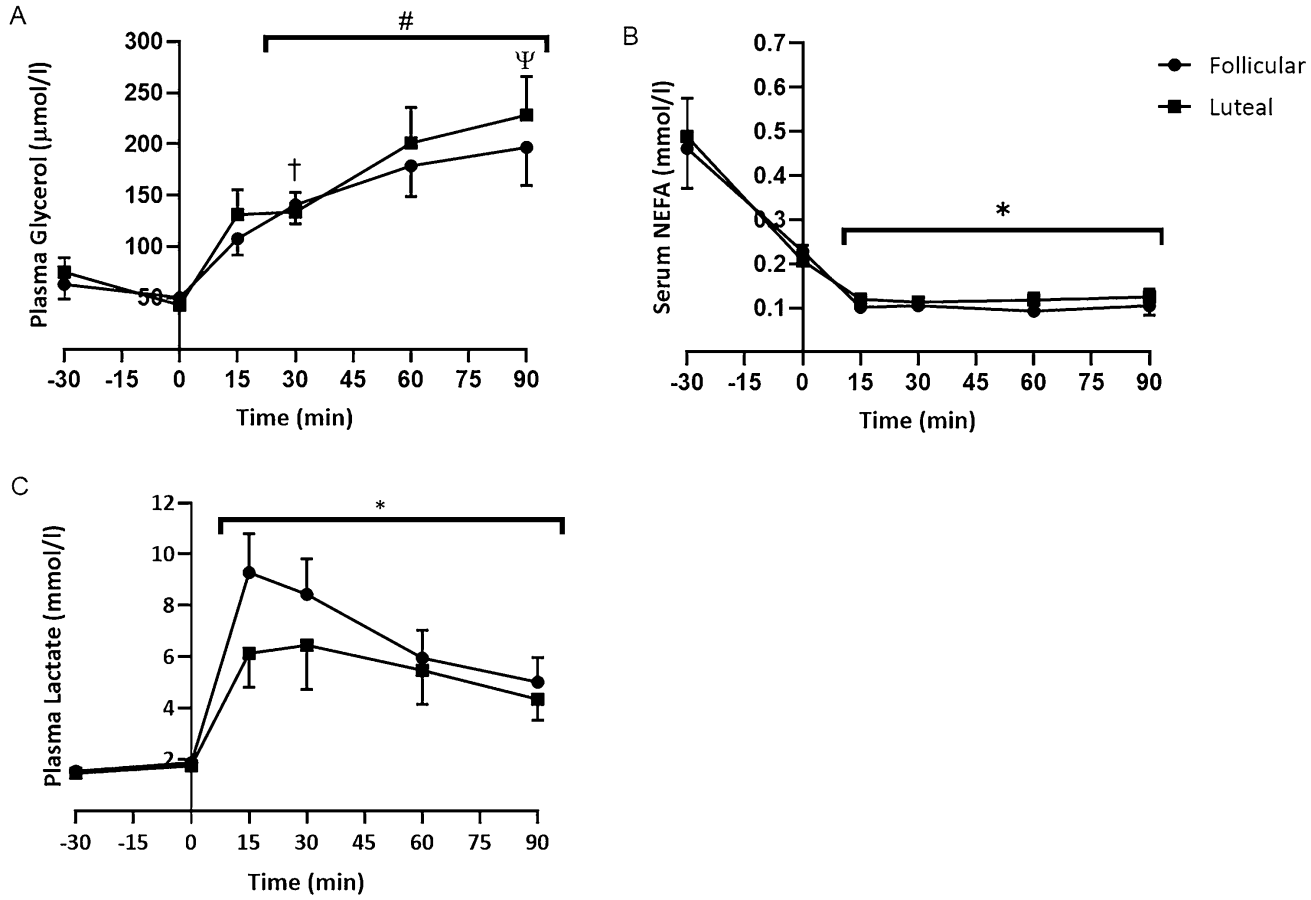
Mean (± SEM) **A** Plasma glycerol, **B** serum NEFA and **C** plasma lactate concentration during the follicular and luteal Phases. *significantly lower than at − 30 min, #significantly greater than at − 30 and 0 min, †significantly greater than at 15 min, ψ significantly greater than at 30 min, **significantly greater than at − 30 and 0 min

Mean serum NEFA concentration significantly decreased from 0.48 ± 0.76 at pre-infusion to 0.22 ± 0.03 mmol/l at 30 min post-infusion (*p* < 0.05), continuing to decrease during the first fifteen minutes of exercise. However, there were no significant differences between the phases. Mean plasma glycerol concentration decreased slightly from 69.32 ± 11.46 µmol/l pre-infusion to 50.70 ± 9.70 µmol/l, 30 min post-infusion, although this did not reach significance. Following 30 min of exercise, the mean plasma glycerol of both phases had significantly increased to 117.53 ± 11.44 µmol/l (*p* < 0.05), that continued to increase to 212.63 ± 25.18 µmol/l at 90 min (*p* < 0.05). However, there were no significant differences between the phases (Fig. 1).

Mean plasma lactate concentration significantly increased at the start of exercise from 1.87 ± 0.14 mmol/l to 9.28 ± 1.50 mmol/l and 1.75 ± 0.14 mmol/l to 6.13 ± 1.32 mmol/l for the follicular and luteal phase and, respectively (*p < *0.01). Mean lactate concentration then decreased throughout the 90 min of exercise to 4.68 ± 0.72 mmol/l. Throughout exercise mean lactate concentration was greater in the follicular phase, although this did not reach significance (*p = *0.07). There was no significant interaction between time and phase.

### Hormones

**Fig. 2:**
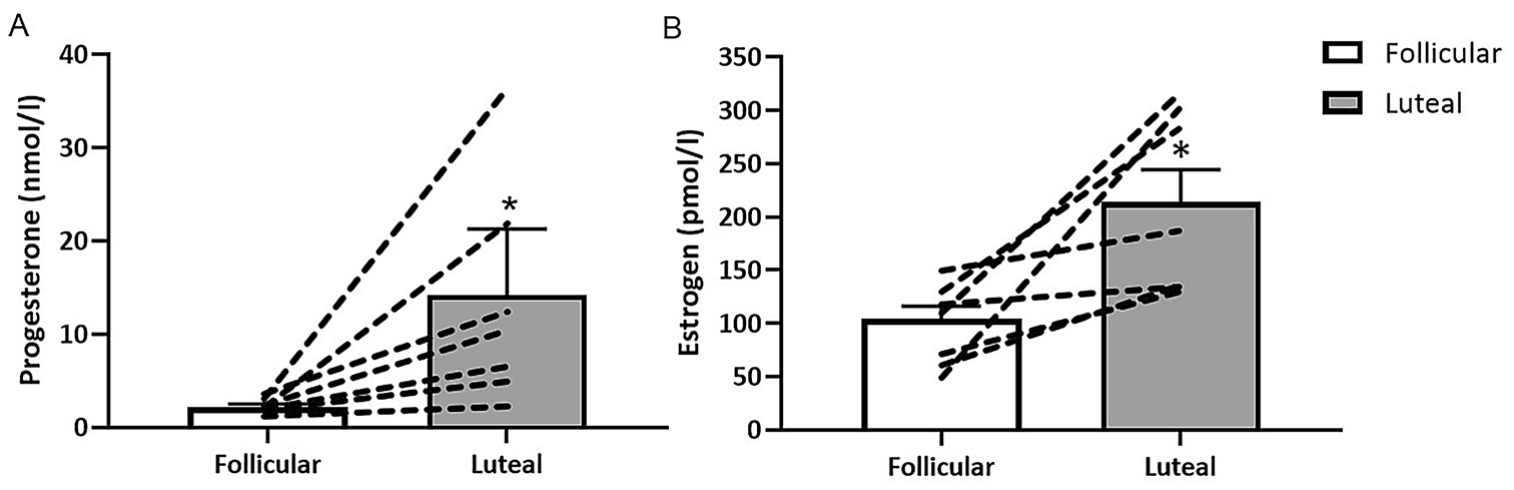
Mean (± SEM) serum progesterone and estrogen concentrations. *significantly greater than in the follicular phase *p < *0.05

Serum estrogen was significantly higher in the luteal phase of the menstrual cycle (lut 213.28 ± 30.70 pmol/l vs foll 103.86 ± 13.85 pmol/l, *p = *0.016CI._95_ -2.25–0.22), as was progesterone (lut 14.23 ± 4.88 nmol/l vs foll 2.11 ± 0.32 nmol/l, *p = *0.042, CI._95_ -1.87–0.04). Cohen’s effect size illustrated a strong effect with values of *d = *1.34 and *d = *0.94 for estrogen and progesterone, respectively, during the luteal phase compared to the follicular phase (Fig. 2).

**Fig. 3:**
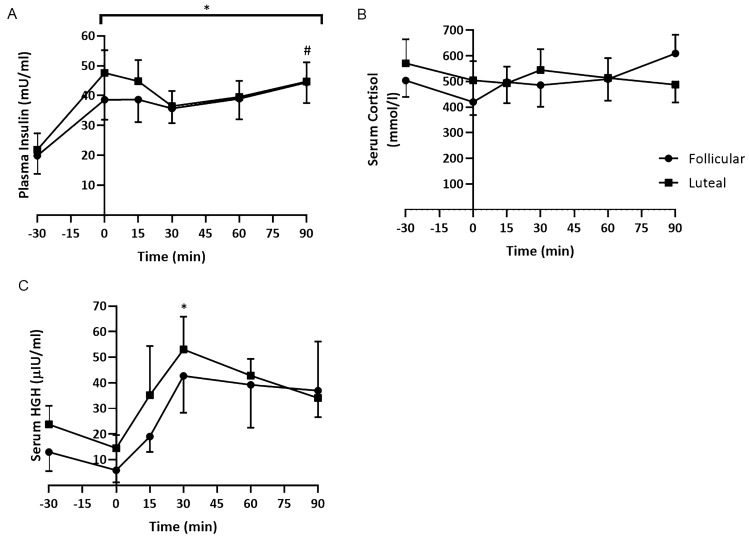
Mean (± SEM) **A** serum insulin concentration, **B** serum cortisol and **C** HGH concentration during the follicular and luteal phases. *significantly greater than at − 30 min, #significantly greater than at 30 min, †significantly greater than at 0 min

The mean serum insulin concentration (Fig. 3) for both phases increased significantly as a result of the glucose infusion from 20.88 ± 5.57 mU/ml to 43.14 ± 6.48 mU/ml (*p < *0.05). During the first 30 min of exercise, there was a slight decrease in insulin concentration to 36.1 ± 4.7 mU/ml, whereupon insulin increased significantly to 44.56 ± 5.06 mU/ml at the end of the exercise. There were no significant differences between the phases.

There was no significant change over time or between the phases for serum cortisol concentration. However, there was a decrease in HGH as a result of the glucose infusion at rest from 18.37 ± 6.76 μlU/ml to 10.16 ± 4.49 μlU/ml that then significantly increased to a peak of 47.91 ± 7.3 μlU/ml (*p < *0.05) at 30 min irrespective of phase. There were no significant differences between the phases.

### Glucose utilisation and substrate oxidation

**Fig. 4:**
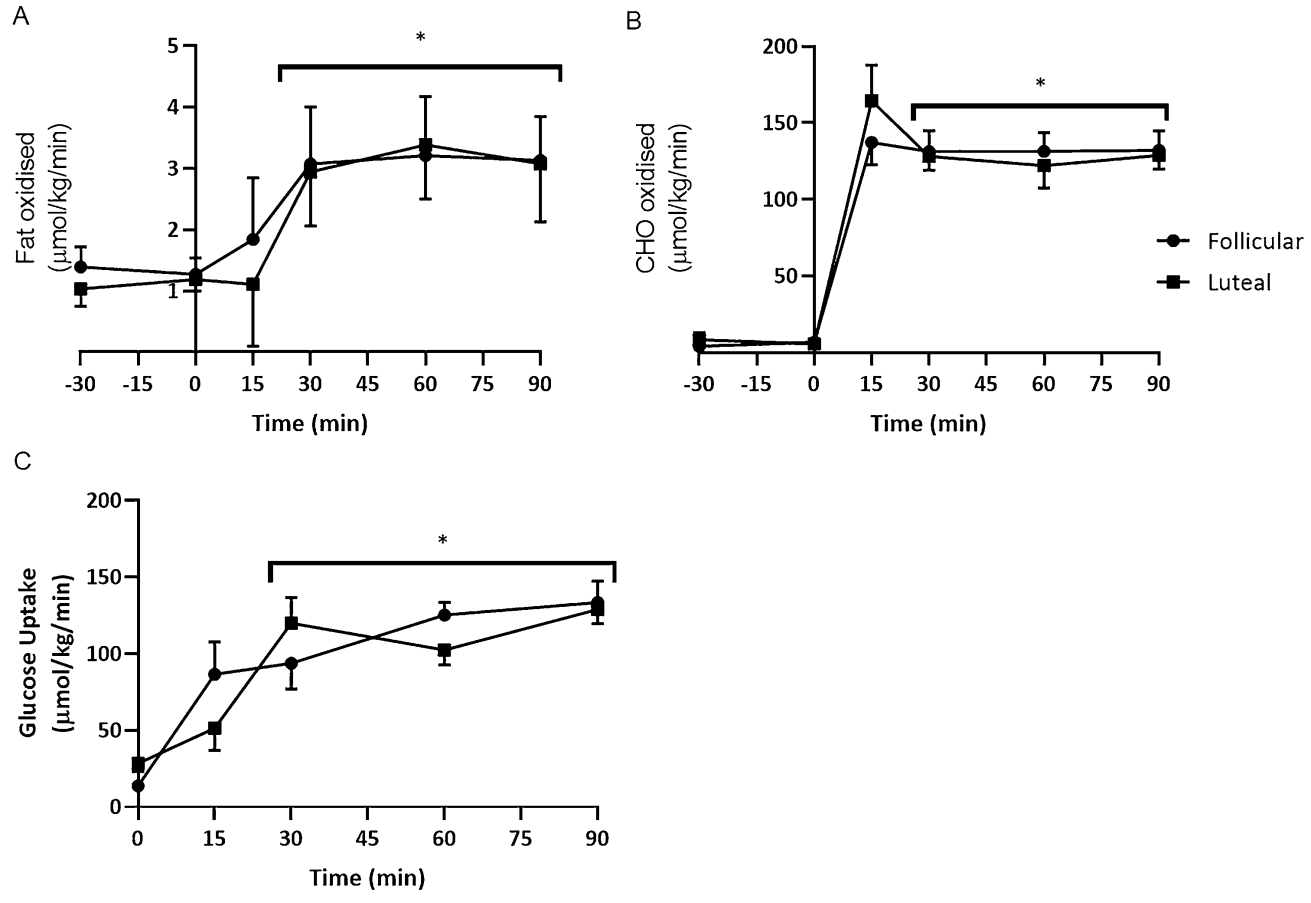
Mean (± SEM) **A** lipid oxidation rate **B** carbohydrate oxidation rate and **C** glucose utilisation rate during the follicular and luteal Phases. *significantly greater than at − 30 and 0 min, **significantly greater than at 0 and 15 min

Glucose utilisation rate, as calculated from the glucose infusion rate, significantly increased during the first 30 min of exercise from 21.04 ± 1.96 μmol/kg/min to 106.83 ± 8.35 μmol/kg/min (*p < *0.05) and continued to increase, peaking at 131.11 ± 10.69 μmol/kg/min at the end of exercise. There were no significant differences between the phases. The glucose infusion had no effect on RER over the first 30 min but exercise resulted in a significant increase from 0.79 ± 0.02 to 0.97 ± 0.01 as confirmed by the significant increase in carbohydrate oxidation from 6.19 ± 1.21 μmol/kg/min at rest to 150.78 ± 6.3 μmol/kg/min after fifteen minutes of exercise (*p < *0.05). Carbohydrate oxidation rate plateaued throughout the rest of the exercise period. Lipid oxidation also significantly increased from 1.23 ± 0.16 μmol/kg/min at rest to 3.00 ± 0.65 μmol/kg/min at 30 min of exercise (*p < *0.05) and then plateaued. There were no significant differences between the phases (Fig. 4).

**Fig. 5:**
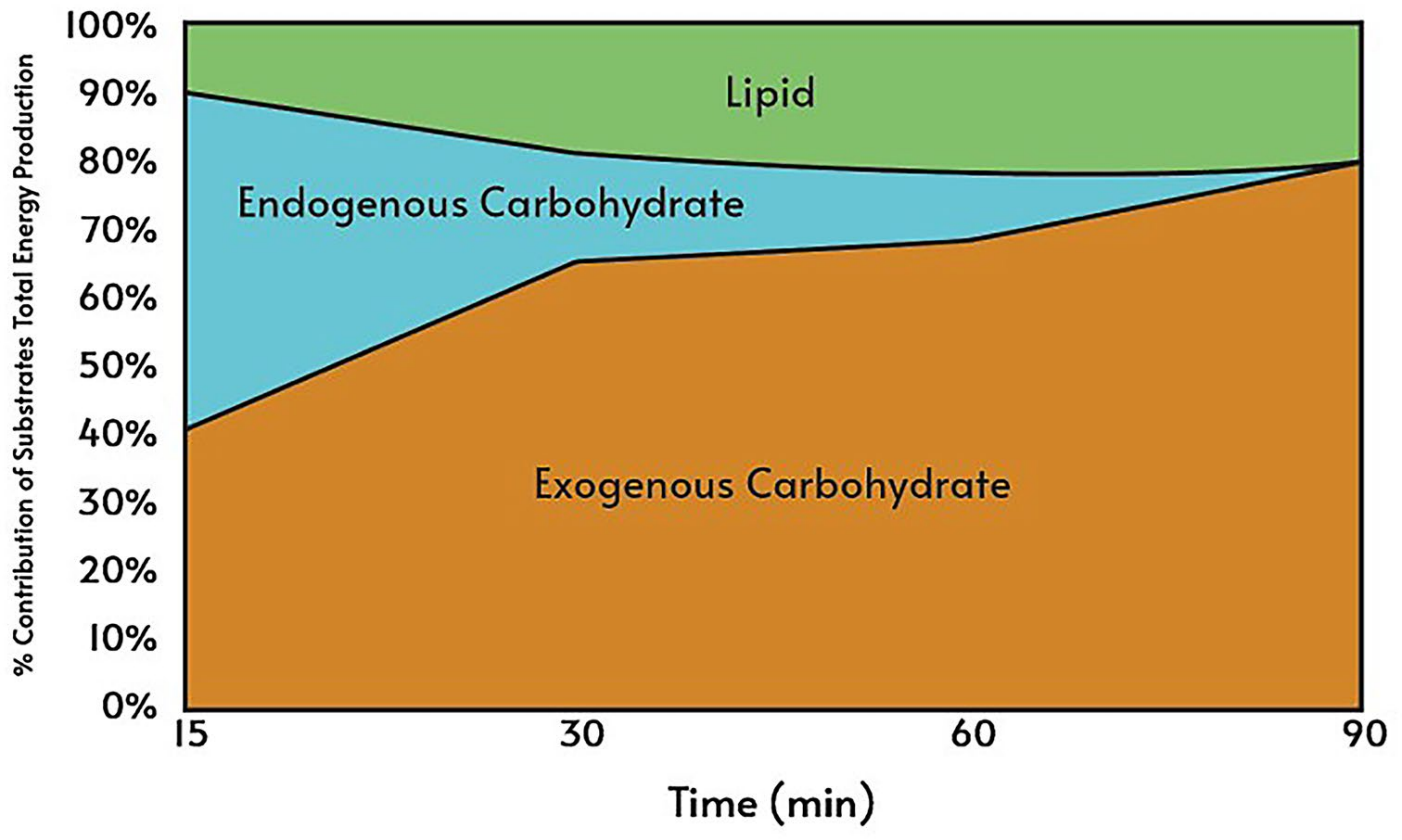
The percentage contribution of substrates to total substrate oxidation using pooled data from both phases

Figure 5 is a schema based on the substrate oxidation findings to show that at fifteen minutes of exercise exogenous carbohydrate contributed ~ 40% of total energy production, and that increased to ~ 65% at 30 min. Furthermore, by 90 min of exercise exogenous carbohydrate was the sole source of carbohydrate oxidation, i.e. no apparent endogenous carbohydrate was being used. Throughout the 90 min, the total contribution of carbohydrate to total energy production decreased (from 90 to 80%), while lipid contribution increased (from 10 to 20%).

## Discussion

This investigation examined glucose metabolism, as well as hormonal and substrate utilisation, in eumenorrheic women during maintained hyperglycaemia throughout submaximal endurance exercise, at two distinct phases of the menstrual cycle. Apart from the anticipated difference in levels of estrogen and progesterone during the luteal phase, which observed a twofold increase in estrogen (Jurkowski et al. 1991; Campbell et al. 2001), the results show no further significant differences in any of the hormones, metabolites or substrate utilisation patterns between phases during maintained hyperglycaemia. These findings support our hypotheses and previous findings (Horton et al. 2002; Kraemer et al. 2013). The low number of participants recruited for this investigation is a limitation and, therefore, caution should be exercised when interpreting the findings.

D'Eon (2002) proposed that a metabolic response to changes in the ovarian hormones can occur when the magnitude of the increase in estrogen from the early follicular phase to comparison phases such as the late follicular or mid-luteal is at least in the order of a twofold increase, such as seen in the present study. The metabolic effects of estrogen have received much interest due to its potential glycogen sparing effect as a consequence of an increased reliance on fat oxidation during the luteal phase, although no metabolic shift was evident in the present investigation. A potential reason for such findings is likely due to the infusion of glucose, which is in line with the results from previous investigations (Bailey et al. 2000; Campbell et al. 2001; Suh et al. 2002), which observed minimal effects to exercise metabolism from elevated levels of estrogen and progesterone following ingestion of CHO throughout the exercise. Indeed, Campbell et al. (2001) further suggest that the ovarian hormones are capable of modifying exercise metabolism, but only in the absence of CHO ingestion, as estrogens have the potential to act as a potent promoter of lipid oxidation. Therefore, by ensuring adequate CHO is provided exogenously during exercise, any variation in exercise metabolism or substrate oxidation induced by a phasic shift is reduced or diminished. Suh et al. (2002) stressed that the metabolic and glucose flux rate between phases were small, and potentially easily overridden by factors such as exercise and the consumption of CHO. However, this assumption was only supported with the addition of CHO and not exercise alone (Bailey et al. 2000; Campbell et al. 2001). Even by employing an exercise intensity of 70% $\dot V{O_{2peak}}$ that may be governed by a glycolytic flux, it was only the addition of CHO that further increased the dependency on CHO oxidation, and overcoming the hormonal changes between phases shown to promote lipid oxidation. Moreover, lower exercise intensities of 60% and 65% $\dot V{O_{2peak}}$ conducted within the current investigation and Suh et al. (2002), respectively, concur with the diminished effect of the ovarian hormones with the presence of CHO.

An elevated concentration gradient for glucose following CHO feeding results in increased plasma glucose levels (Campbell et al. 2001), this may explain the diminished effect of the ovarian hormones on metabolism. Therefore, glucose transport is unaffected by ovarian hormones during exogenous CHO intake, with significantly lower plasma glucose observed during control trials emphasising the importance of glucose transport capacity. Furthermore, Campbell et al. (2001) affirm that the glucose rate of disappearance is increased during CHO ingestion during exercise, in addition to augmented oxidation of plasma glucose that may potentially spare glycogen. However, the sparing of glycogen is not universally accepted during CHO feeding (Coyle et al. 1986), and may suggest a sex difference as a greater glucose flux in women has been reported (Friedlander et al. 1998). Further, it has been proposed that females have a greater capacity to utilise glucose during exercise and receive a greater advantage from CHO ingestion than previously seen in male counterparts (Campbell et al. 2001). It should be noted that Campbell et al. (2001) only used a female cohort so a direct comparison with males was unavailable. Having said that, conflicting evidence is proposed when direct comparisons are made between males and females (Wallis et al. 2006). In their investigation, Wallis et al. (2006) found no differences in CHO oxidation of ingested CHO during exercise. Nevertheless, the current investigation supports the notion that females may have a greater capacity, as it is clear that by infusing CHO, the utilisation of the exogenous CHO increases throughout the exercise and so is likely to spare muscle glycogen irrespective of the menstrual cycle phase. Figure 5 clearly illustrates that there is no endogenous CHO oxidation at 90 min.

Mean blood glucose concentration (10.1 ± 0.1 mmol/l) remained tightly controlled during the hyperglycaemic clamp as demonstrated by the low standard error and highlights the suitability of the clamp for examining glucose metabolism during exercise. Further, the maintenance of mean heart rate throughout exercise demonstrated that exercise intensity remained constant throughout the exercise period. The infusion resulted in an immediate decrease in circulating NEFA indicating a decrease in lipolysis due to the hyperglycaemic and hyperinsulinaemic stimulus. Insulin is an antilipolytic hormone that decreases the rate of lipolysis, in part, by decreasing the activity of hormone-sensitive lipase. Glycerol concentration also decreased at rest due to the glucose infusion, although throughout exercise there was a significant increase. During exercise when hyperglycaemic, previous studies have reported significant increases in adrenaline and noradrenaline (MacLaren et al. 1999), both lipolytic hormones. Therefore, the increase in glycerol concentration during exercise reflects an increase in lipolysis, although the decrease in NEFA shows there is re-esterification of NEFA at a greater rate than its production. This is in agreement with Coyle et al. (1991) who found that plasma NEFA concentration was suppressed during hyperglycaemia compared to euglycaemia and suggested this was due to insulin-induced acceleration of adipocyte triglyceride resynthesis. Low circulating NEFA concentration during hyperglycaemia and increased insulin concentration also facilitate a high rate of glucose disposal by exercising muscle (Rennie and Holloszy 1977).

Previous hyperglycaemic clamp studies using labelled glucose have demonstrated that when blood glucose is maintained at 10 mmol/l hepatic glucose production is completely suppressed (Hawley et al. 1994). In our study, GUR increased throughout exercise, as has been reported in hyperglycaemic studies using males. This increase is likely due to increased translocation of GLUT4 transporters to the cell membrane, insulin-stimulated increase in blood flow to the muscle, and activation of hexokinase activity within muscle (Malone et al. 2021). Coyle et al. (1991) and Hawley et al. (1994) found glucose infusion rates to increase throughout exercise and reported infusion rates of about 2.3 g/min and 1.22 g/min at 90 min of exercise, respectively. One study that used a similar protocol using male participants (MacLaren et al. 1999) had a 90-min GUR of 1.8 g/min, compared to 1.44 g/min observed in the present study. However, MacLaren et al. (1999) clamped blood glucose at 12 mmol/l and the exercise was at a higher intensity of 70% $\dot V{O_{2max}}$; factors likely to explain the higher GUR. Serum insulin concentration remained lower than that reported in the present study (~ 30 mU/l vs 45 mU/l at 90 min) possibly due to the lower intensity and followed a different pattern. In males, insulin has been reported to peak during exercise with the hyperglycaemic clamp at between 60 and 80 min and then decrease (Coyle et al. 1991; MacLaren et al. 1999). Increased adrenaline concentration inhibiting insulin secretion has been the proposed mechanism with a possible threshold of 50% above resting values. In the present study, adrenaline was not measured but it is possible that adrenaline concentration did not increase to a large enough degree to reach the ‘threshold’, and this is supported by evidence that there is a lower catecholamine response to stress in females compared to males (Horton et al. 1998).

During the hyperglycaemic clamp, when hepatic glucose production is completely suppressed and glucose utilization rate is determined by the glucose infusion rate, endogenous carbohydrate utilization can be estimated. Total carbohydrate oxidation rate minus GUR is an indication of endogenous carbohydrate utilization (Fig. 5). Throughout exercise, the percentage lipid contribution to total substrate oxidation increased from 10 to 20% whereas carbohydrate contribution decreased from 90 to 80%. These percentage contributions of lipid oxidation are similar to previously reported values of between 12 and 22% (Hawley et al. 1994; Weltan et al. 1998). Furthermore, a greater contribution of endogenous carbohydrate to total carbohydrate oxidation is observed at the start of exercise and throughout the first 60 min than previously reported. The current investigation administered a prime infusion of glucose for 30 min at rest, increasing both glucose and insulin concentrations pre-exercise, whereas the earlier studies started the infusion and exercise simultaneously. This would have resulted in increased glucose availability pre-exercise and therefore favoured blood glucose oxidation over muscle glycogen utilisation. Indeed, during the first 60 min of exercise, CHO oxidation exceeded glucose utilisation and endogenous store was utilised. Previous studies in males have also found that the total carbohydrate oxidation rate exceeds GUR resulting in endogenous carbohydrate oxidation (Hawley et al. 1994; Weltan et al. 1998). These are supported by studies that found that hyperglycaemia does not alter net glycogen utilization in males (Coyle et al. 1991; MacLaren et al. 1999). At 75 min of exercise, GUR was slightly greater than carbohydrate oxidation, suggesting non-oxidative glucose disposal such as glycogen storage, or triglyceride formation.

Findings of non-oxidative glucose disposal in the present investigation are in keeping with the results from Mohebbi et al. (2020) and can be attributed to the suppression of endogenous glucose production, which reflects a trend towards conserving muscle glycogen stores. However, Mohebbi et al. (2020) performed both a hyperglycaemic and hyperinsulinemic clamp that may have augmented exogenous sources. When similar hyperglycaemic clamps in the absence of insulin infusion have been conducted (Maclaren et al. 1999; Malone et al. 2020) non-oxidative glucose disposal has not been evident, thus supporting the assumption that females’ favour greater lipid oxidation (Hackney et al. 1994) and less CHO oxidation resulting in increased storage, although it must be noted that the exercise intensity was lower in the current investigation. Therefore, whether these patterns are due to differences in protocols or sex is yet to be confirmed. One possible mechanism could be the increased insulin concentration observed in the present study compared to others (Maclaren et al. 1999; Malone et al. 2020), as hyperinsulinemia accelerates glucose transport and hexokinase activity, which in turn increases intracellular glucose 6-phosphate concentration promoting glycogen synthesis. In addition, Donahue et al. (1996) found that insulin sensitivity differs between sexes and that glucose uptake at a constant insulin level was greater in females.

The suppression of growth hormone concentration at rest as a result of the hyperglycaemia is probably a result of a decrease in activation of α-receptors by the cells of the hypothalamic ventromedial nuclei that are gluco-receptors (Hansen 1971). Similarly, the slight decrease in cortisol concentration at rest is also possibly due to gluco-receptors supported by evidence that a high-fat diet increases cortisol release and high-carbohydrate diet decreases cortisol release (Galbo et al. 1979; Venkatraman et al. 2001). Conversely, the increase in HGH concentration during exercise is due to impulses from the brain and active muscles causing an increase in sympathoadrenal activity and increased release of both HGH and ACTH. The elevation in ACTH also results in increased cortisol release, supporting the slight increase seen over the exercise period.

In conclusion, this was the first study to examine glucose utilization in females during exercise using the hyperglycaemic clamp method. The main finding was that, in spite of significantly elevated levels of estrogen during the luteal phase, hyperglycaemia maintained during exercise did not result in differences in hormonal and metabolic responses between phases of the menstrual cycle. Differences were noted for rates of glucose utilization, carbohydrate oxidation and lipid oxidation that had previously been reported using males, although this may be due to the lower exercise intensity employed in the present study. These findings support previous investigations (Bailey et al. 2000; Campbell et al. 2001) and provide practical implications advocating the consumption of CHO during endurance exercise due to its ability to diminish any metabolic variations incurred by the menstrual cycle.

## Abbreviations

ACTH: Adrenocorticotropic hormone
CHO: Carbohydrate
CV: Co-efficient of variation
ELISA: Enzyme-linked immunosorbent assay
GLUT4: Glucose transporter 4
GUR: Glucose utilisation rate
HGH: Growth hormone
NEFA: Non-esterified fatty acid
RER: Respiratory exchange ratio
$$\dot V{O_{2}}$$: Oxygen consumption
$\dot V{O_{2peak}}$: Peak oxygen consumption
$\dot V{O_{2max}}$: Maximal oxygen consumption
